# Allergenic activity and ability to induce T cell and cytokine responses of different infant milk formulas

**DOI:** 10.1186/1939-4551-8-S1-A253

**Published:** 2015-04-08

**Authors:** Heidrun Hochwallner, Ulrike Schulmeister, Udo Herz, Margarete Focke-Tejkl, Ines Swoboda, Renate Reininger, Vera Civaj, Raffaela Campana, Josef Thalhamer, Sandra Scheiblhofer

**Affiliations:** 1 Division of Immunopathology, Department of Pathophysiology and Allergy Research, Medical University of Vienna, Austria; 2 Department of Medical & Chemical Laboratory Diagnostics, Medical University of Vienna, Austria; 3 Mead Johnson Nutrition Gmbh, Evansville, Germany; 4 Christian Doppler Laboratory for Allergy Diagnosis & Therapy, Department of Molecular Biology, University of Salzburg, Austria

## Background

Many hydrolyzed cow’s milk (CM) formulas are available for avoidance of allergic reactions in cow’s milk allergic children and for prevention of allergy development in high risk infants.

CM formulas were compared regarding the presence of immunoreactive CM components, IgE reactivity, allergenic activity, ability to induce T cell proliferation and allergic or pro-inflammatory cytokine secretion.

## Methods

Using biochemical techniques and antibody probes highly specific for seven different cow’s milk allergens, a blinded analysis of a panel of eight cow’s milk formulas, one non-hydrolyzed, two partially hydrolyzed, four extensively hydrolyzed and one amino acid formula, was conducted. IgE reactivity and allergenic activity of the formulas were tested with sera from cow’s milk allergic patients (n=26) in RAST-based assays and with rat basophils transfected with the human FcεRI, respectively. Furthermore, the induction of T cell proliferation and the secretion of a panel of cytokines in PBMC cultures from cow’s milk allergic patients and non-allergic individuals were assessed.

## Results

Immune-reactive whey proteins (alpha-lactalbumin, beta-lactoglobulin) were found in the two partially hydrolyzed formulas and casein components in one of the extensively hydrolyzed formulas. One partially hydrolyzed formula and the extensively hydrolyzed formula containing casein components showed remaining IgE reactivity whereas the other hydrolyzed formulas lacked IgE reactivity. Interestingly, only two extensively hydrolyzed formulas and the amino acid formula did not induce T cell proliferation and pro-inflammatory cytokine release whereas the remaining formulas varied regarding the induction of Th2, Th1 and pro-inflammatory cytokines.

## Conclusions

The investigated CM formulas showed a great variability regarding the presence of immunogenic CM components, IgE reactivity, allergenic activity and induction of pro-inflammatory cytokines. These results of our study may explain different outcomes obtained in clinical studies using CM formulas for prevention and treatment and they show that certain CM formulas without allergenic and low pro-inflammatory properties can be identified.

